# A Dispersion Compensation Method Based on Resampling of Modulated Signal for FMCW Lidar

**DOI:** 10.3390/s21010249

**Published:** 2021-01-02

**Authors:** Shuo Jiang, Bo Liu, Shengjie Wang

**Affiliations:** 1 Key Laboratory of Science and Technology on Space Optoelectronic Precision Measurement, CAS, Chengdu 610200, China; jiangshuo15@mails.ucas.ac.cn (S.J.); wangshengjie@cafuc.edu.cn (S.W.); 2 Institute of Optics and Electronics, Chinese Academy of Sciences, Chengdu 610200, China; 3 University of Chinese Academy of Sciences, Beijing 101400, China; 4 Civil Aviation Flight University of China, Deyang 618300, China

**Keywords:** FMCW laser ranging, fiber dispersion mismatch, dispersion compensation, resampling

## Abstract

In order to eliminate the nonlinearity in the laser modulation process, the dual-interferometers system is often adopted in the frequency modulation continuous wave (FMCW) laser ranging. However, the dispersion mismatch between the fiber reference interferometer and the measurement interferometer will lead to the decrease in ranging accuracy and resolution. In this paper, a dispersion compensation method based on resampling with a modulated signal is proposed. Since the beat signal of the end face of the delay fiber is not affected by dispersion mismatch, it can be modulated to generate a signal whose phase is proportional to that of the target spatial signal. Then, the modulated signal is regarded as the reference clock to sample the target spatial signal. Thereby, the influence of the dispersion mismatch between the two optical interferometers can be eliminated. In this article, simulation is performed to verify the effect of this method, and an experiment is carried out on the target at the distance of 2.4 m. Experiments show that the full width at half maximum (FWHM) of the distance spectrum after dispersion compensation is consistent with the reflected signal from the end face of the delay fiber, and the standard deviation of multiple measurements reached 10.12 μm.

## 1. Introduction

FMCW laser ranging is an active detection system that takes the laser with linear modulated frequency as the light source to measure the target distance. The target distance can be obtained by measuring the frequency of the beat signal in measurement interferometer [1,2]. With the development of laser modulation technology, the modulation bandwidth of the light source gradually improves from several GHz to THz, which makes the ranging resolution reach the micron scale. In addition, it also has the advantages of no ranging blind area and high ranging accuracy, making FMCW lidar widely used in high-precision target 3D coordinate measurement, precision instrument processing, and other fields [3,4,5,6,7].

Due to the influence of internal mechanical control accuracy, nonlinear creep, and the hysteresis effect, ideal linear modulation is hard to achieve in the actual modulation process, which will result in a serious reduction in the precision and resolution of ranging [8]. In order to solve this problem, a fiber reference interferometer is introduced into the system. Since the measurement interferometer and the reference interferometer have the same light source, the effect of the laser nonlinear modulation on the two beat signals is consistent. Resampling the measurement signal by taking the reference signal as the external clock can eliminate the nonlinear influence [9]. However, the dispersion mismatch between the fiber reference interferometer and the measurement interferometer will lead to the decrease in ranging accuracy and resolution. The traditional solution is to introduce a dispersion-balanced material into the interference arm of the interferometer. Nevertheless, this method will increase the complexity of the system [10]. To reduce the system requirement on dispersion compensation, more and more numerical dispersion compensation methods have been studied. In 2012, Norman used the fractional Fourier transform (FrFT) to solve the problem of dispersion mismatch, which was introduced by a 26 mm long water cell and obtained the range resolution of 3.6 μm with the modulation bandwidth of 110 nm [11]. In 2015, Lu proposed a dispersion mismatch compensation algorithm based on chirp decomposition; the experiment measured a target of 3.9 m with the modulation bandwidth of 4.26 THz and achieved the range resolution of 45.9 μm [12]. In 2018, Shi proposed an FMCW laser ranging system based on dispersion phase compensation. In the experiment, the modulation bandwidth was set to 10 nm and the target of 65 m was measured with the resolution of 123 μm [13]. In this article, a dispersion compensation method based on resampling with modulated signal is proposed. In the measurement interferometer, because the signal reflected from the fiber end face is not affected by dispersion mismatch, it can be modulated to generate a new signal whose phase is proportional to that of the target spatial signal. Taking the modulated signal as the reference and sampling the target spatial signal with equal phase intervals, then the dispersion mismatch between the two interferometers can be eliminated. 

## 2. Theoretical Principles and Models 

### 2.1. The Ranging Process of FMCW Lidar

**Figure 1:**
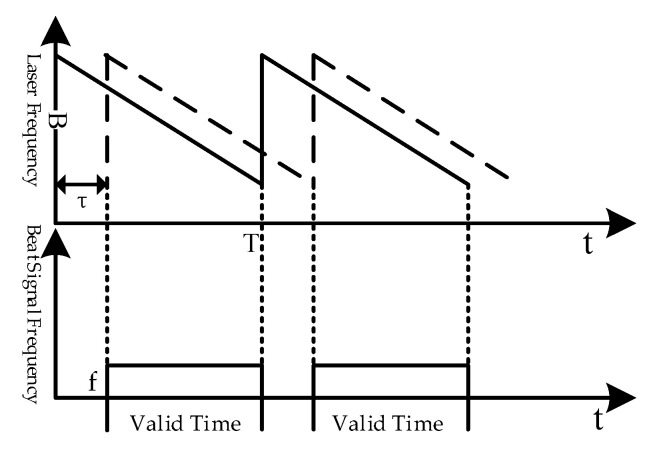
The ranging principle diagram of frequency modulation continuous wave (FMCW) lidar.

As shown in Figure 1, the solid line represents the instantaneous frequency of the local oscillator laser, and the dashed line represents the frequency of the reflected laser from the target. B is the modulation bandwidth (the optical frequency difference corresponding to the start and end wavelengths of the laser), T is the modulation period, and τ is the time delay between the two lasers. Beat signals are obtained coherently by the two lasers on a detector, and the frequency of the beat signal is f.

Ideally, the frequency of beat signals is proportional to the target distance. According to the principle of similar triangles, the distance of the target can be expressed as:

(1) $$R_{m}=\frac{Tc}{2B}\cdot f$$

where $R_{m}$ stands for the target distance and c stands for the speed of light.

Affected by the laser nonlinear modulation, the frequency of the actual beat signal is not fixed but rather a variable value, which greatly reduces the resolution and accuracy of the system. The dual-interferometers system is often adopted to deal with this issue for the phases of the beat signals in the two interferometers are proportional. Through sampling the measurement signal at every valley and peak point of the reference signal, the influence of nonlinear modulation of the laser can be solved [8]. The expression of the resampling signal can be obtained as follows:

(2) $$I(n)=A\cos(2\pi \frac{\tau_{m}}{2\tau_{Ref}}\cdot n)$$

where A represents the signal amplitude, $\tau_{m}$ and $\tau_{Ref}$ represent the time delay of the measurement and reference interferometers, and n stands for the number of the resampling points.

It can be seen from Equation (2) that the frequency of the resampling signal is fixed, and the effect of laser modulation nonlinearity on ranging can be eliminated. Then, the target distance can be obtained.

### 2.2. Influence of Optical Fiber Dispersion of Reference Interferometer on Ranging

In the dual-interferometers system, a fiber Mach–Zehnder interferometer is often added as the reference interferometer. In the single-mode fiber, the laser group velocity is a function related to the wavelength. When the frequency-modulated laser passes through a delay fiber, the time delay of each frequency component is no longer a constant. Using the beat signal of the reference interferometer as the system clock will cause the broadening and deviation of the target distance spectrum.

In the fiber reference interferometer, the beat signal is expressed as:

(3) $$I_{Ref}(t)=A_{Ref}\exp\left[-i(\frac{2\pi \mu \beta_{1}x}{1+2\pi \mu x\beta_{2}}t+\frac{{\left(2\pi \mu \right)}^{2}\beta_{2}x}{2(1+2\pi \mu x\beta_{2})}t^{2})\right]$$

where $A_{Ref}$ represents the amplitude of the reference signal, μ represents the instantaneous modulation speed of the laser, x is the length of the delay fiber, $\beta_{1}$ is the reciprocal of the group velocity (β1 = 1/vg), and $\beta_{2}$ is the fiber dispersion coefficient.

According to Equation (3), the time delay of the modulated laser passing through the fiber reference interferometer can be obtained:

(4) $$\tau_{Ref}(t)=\frac{x(\beta_{1}+\pi \mu \beta 2t)}{1+2\pi \mu x\beta_{2}}=\frac{\tau_{Ref}(1+\pi \mu \beta_{2}v_{g}t)}{1+2\pi \mu \beta_{2}v_{g}\tau_{Ref}}$$

where $\tau_{Ref}$ represents the time delay of the laser with the initial wavelength passing through the fiber.

At present, a G. 652 D standard single-mode fiber is the most widely used single-mode fiber. When $\beta_{2}=-23\times 10^{-27}s^{2}/m$, $v_{g}=2.0437\times 10^{8}m/s$, x is 50 m and μ=10THz/s, and it can be calculated that $1+2\pi \mu \beta_{2}v_{g}\tau_{R}{}_{ef}\approx 1$. Therefore, Equation (4) can be simplified to:

(5) $$\tau_{Ref}(t)=\tau_{Ref}(1+\pi \mu \beta_{2}v_{g}t)$$

Resampling the measurement signal at the valley and peak of the reference signal, the relationship between t and n can be derived from Equation (3). Expression of the resampling target signal that is affected by the dispersion mismatch can be acquired:

(6) $$\begin{array}{ll} I_{m}(n) & =A_{m}\exp\left[-i(2\pi \frac{\tau_{m}}{2\tau_{Ref}(t)}\cdot n)\right]\\ & \approx A_{m}\exp\left[-i2\pi (\frac{\tau_{m}}{2\tau_{Ref}}\cdot n-\frac{\tau_{m}\pi \beta_{2}v_{g}}{4\tau_{Ref}^{2}}n^{2})\right] \end{array}$$

where $A_{m}$ is the amplitude of the signal.

Compared with Equation (2), the resampling target signal acquired in Equation (6) has an additional chirped phase that leads to the decrease in ranging resolution.

### 2.3. Dispersion Compensation Method Based on Resampling of Modulated Signal

To deal with the problem, a dispersion compensation method based on resampling with a modulated signal is proposed according to the characteristic of the resampling target signal in Equation (6).

**Figure 2:**
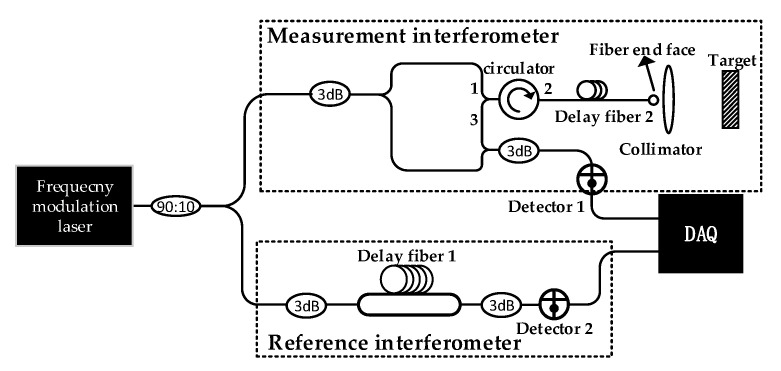
The system diagram of the dispersion compensation method.

As shown in Figure 2, the frequency modulation laser enters the measurement interferometer and reference interferometer, respectively. In the measurement interferometer, the laser goes through a fiber circulator and passes the delay fiber 2; then, it is emitted to the target through the collimator. The reflected laser is recombined with the local oscillator laser to obtain the measurement signal at detector 1. In the reference interferometer, the optical path difference (OPD) is provided by the delay fiber 1, and the beat signal is obtained at detector 2. The two signals are collected by the data acquisition card (DAQ); then, the reference signal is regarded as the system clock to sample the measurement signal with equal phase intervals.

In the measurement interferometer, the components of the measurement resampling signal are mainly composed of the following parts:

1. The resampling signal of the laser leakage from port 1 to 3 of the circulator;
2. The resampling signal of the laser reflected from port 2 to 3 of the circulator;
3. The reflected resampling signal from the end face of delay fiber 2;
4. The resampling target signal.

The first three resampling signals are not affected by the dispersion mismatch, as their propagation paths and the fiber reference interferometer are in the single-mode fiber. However, the target-reflected laser passes through the air, and dispersion mismatch exists between the resampling target signal and the reference signal.

The resampling signal of the delay fiber 2 end face and the resampling target signal are respectively shown as follows:

(7) $$I_{fiber}(n)=A_{fiber}\cos(2\pi \frac{\tau_{fiber}}{2\tau_{Ref}}\cdot n)$$

(8) $$I_{target}(n)=A_{target}\cos(2\pi \frac{\tau_{fiber}+\tau_{air}}{2\tau_{Ref}}\cdot n-\frac{\tau_{air}\pi^{2}\beta_{2}v_{g}}{2\tau_{Ref}^{2}}n^{2})$$

where $\tau_{fiber}$ is the time delay of the end face of the delay fiber 2; $\tau_{air}$ is the time delay of the laser propagating in the air, and $A_{fiber}$ and $A_{target}$ are the amplitudes of the two signals.

The above two signals are obtained from the measurement resampling signal through a linear phase band-pass filter, and the resampling target spatial signal is obtained through phase operation:

(9) $$\begin{array}{cc} I_{air}(n) & =A_{air}\cos(2\pi \frac{\tau_{air}}{2\tau_{Ref}}\cdot n-\frac{\tau_{air}\pi^{2}\beta_{2}v_{g}}{2\tau_{Ref}^{2}}n^{2})\\ & =A_{air}\cos\left[2\pi \frac{\tau_{air}}{2\tau_{Ref}}\cdot n(1-\frac{\pi \beta_{2}v_{g}}{2\tau_{Ref}}n)\right] \end{array}$$

where $A_{air}$ is the signal amplitude. 

By comparing Equation (7) with Equation (9), it can be found that the phase in Equation (9) is proportional to the phase of the following modulated signal:

(10) $$I_{Fiber}(n)=A_{Fiber}\cos\left[2\pi \frac{\tau_{fiber}}{2\tau_{Ref}}\cdot n(1-\frac{\pi \beta_{2}v_{g}}{2\tau_{Ref}}n)\right]$$

where $A_{Fiber}$ stands for the modulated signal amplitude.

Equation (10) is the modulated signal of Equation (7) and the modulation coefficients is $-\pi \beta_{2}v_{g}/2\tau_{Ref}$. Take $I_{Fiber}(n)$ as the new reference clock, and then sample $I_{air}(n)$ at every valley and peak of $I_{Fiber}(n)$, and the target distance in the air can be acquired. The new resampling signal after dispersion compensation can be expressed as:

(11) $$I_{Air}(m)=A_{Air}\cos(2\pi \frac{\tau_{air}}{2\tau_{fiber}}\cdot m)$$

where $A_{Air}$ is the signal amplitude and m is the number of modulated signal resampling.

The modulation coefficient $-\pi \beta_{2}v_{g}/2\tau_{Ref}$ is derived from the evolution of the peak variation distortion elimination method [14]. The method takes the peak amplitude of the target distance spectrum as the judgment criterion to determine the optimal signal modulation coefficient.

## 3. Experimental Results and Analysis

### 3.1. Simulation

Based on Equations (3)–(11), the dispersion compensation method based on the resampling of modulated signals is clarified. Since the frequency of laser modulation is nonlinear, the simulation uses polynomial μ(t) to characterize the instantaneous modulation speed of the laser. The simulation parameters are shown in Table 1.

**Figure 3:**
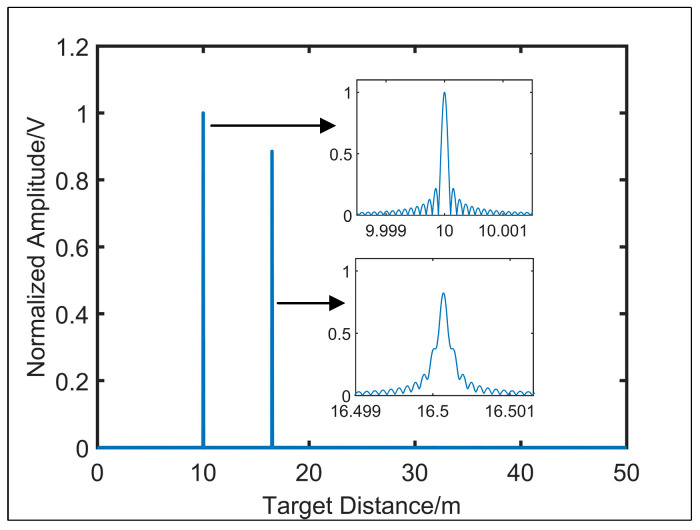
Simulation diagram of the resampling distance spectrum.

In Figure 3, the signal at 16.5 m represents the resampling signal of the target, and the signal located at 10 m represents the end face of delay fiber 2. It can be seen from the simulation figure that the full width at half maximum (FWHM) of the target signal is broadened, and the measured distance deviates from the set distance as a result of the dispersion mismatch.

By using the linear phase band-pass filter, the reflected signal of the delay fiber 2 end face and the target are obtained, respectively. After phase operation, the resampling target spatial signal can be acquired. Then, modulate the signal of the delay fiber 2 end face with the modulation coefficient $-\pi \beta_{2}v_{g}/2\tau_{Ref}$. Sample the resampling target spatial signal at the time points corresponding to the valley-peak of the modulated signal, and the dispersion compensation will be completed.

**Figure 4:**
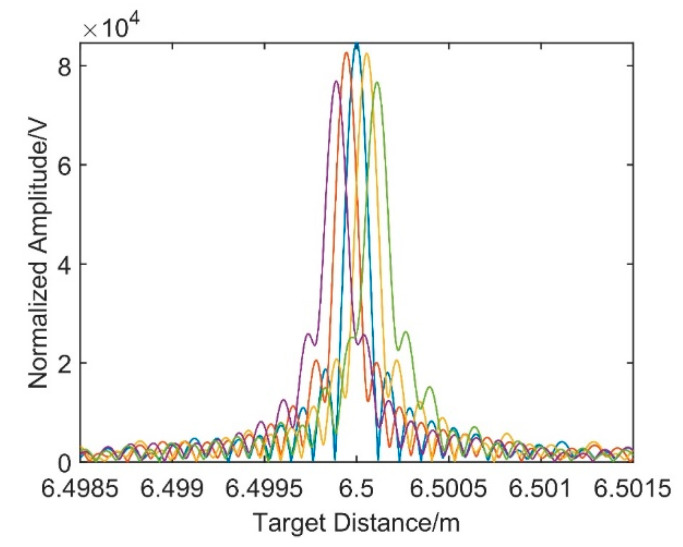
Simulation result based on resampling dispersion compensation method.

Figure 4 shows the dispersion compensation results with five different modulation coefficients where the modulation coefficients of the first and fifth distance spectra are symmetric with that of the third one whose modulation coefficient is $-\pi \beta_{2}v_{g}/2\tau_{Ref}$, and it is the same for the second and fourth distance spectra. The evolution of the peak variation distortion elimination method takes the peak amplitude of the target distance spectrum as the judgment criterion to determine the optimal signal modulation coefficient. The closer the modulation coefficient is to $-\pi \beta_{2}v_{g}/2\tau_{Ref}$, the higher the distance spectrum amplitude. In the third distance spectrum, the broadening and offset caused by dispersion mismatch is eliminated. Simulation results verify the effectiveness of the dispersion compensation method based on the resampling of the modulated signal. 

### 3.2. Experiment

**Figure 5:**
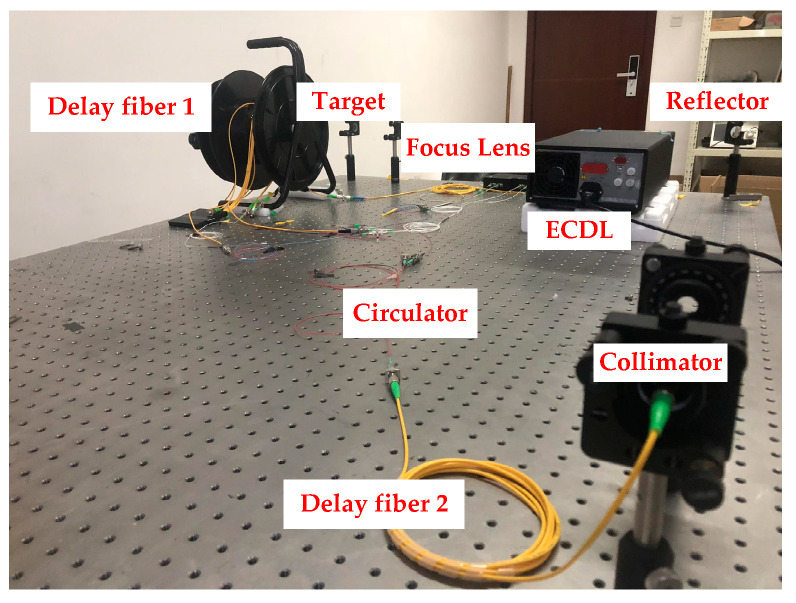
Frequency modulation continuous wave (FMCW) laser ranging system diagram.

In the FMCW laser ranging system, Santec TSL-710 is taken as the modulation laser source, the laser modulation bandwidth is from 1545 to 1565 nm, and the modulation speed is 10 nm/s. In Figure 5, the delay fiber 1 provided the OPD of the reference interferometer. In the measurement interferometer, the delay fiber 2 is connected with the circulator, and then the laser emits to the target through a collimator. Limited by the length of the optical platform, the propagation path in the air is folded into an “L” shape, and a focus lens is put in front of the target mirror to focus the laser. All mirror holders are adjusted and fixed on the optical platform.

**Figure 6:**
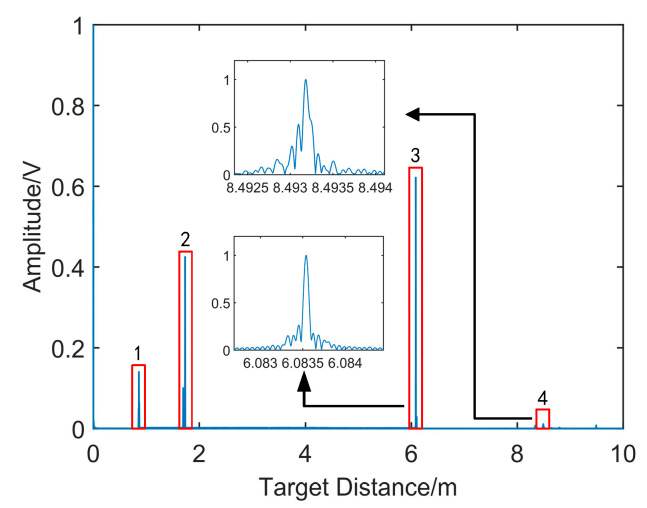
The distance spectra of the resampling signal.

Figure 6 shows the distance spectrum resampling with the reference signal as the system clock. In this figure, signal 1 is the resampling signal of the laser leakage from port 1 to 3 of the circulator; signal 2 is the resampling signal of the laser reflected from port 2 to 3 of the circulator; signal 3 is the reflected resampling signal from the end face of delay fiber 2; and signal 4 is the resampling signal of target. The larger version of signal 3 and 4 is shown in this figure. Compared with the spectrum of signal 3, the spectrum of the target signal is broadened. The FWHM of signal 3 is 73 μm, and the FWHM of signal 4 reaches 112 μm.

**Figure 7:**
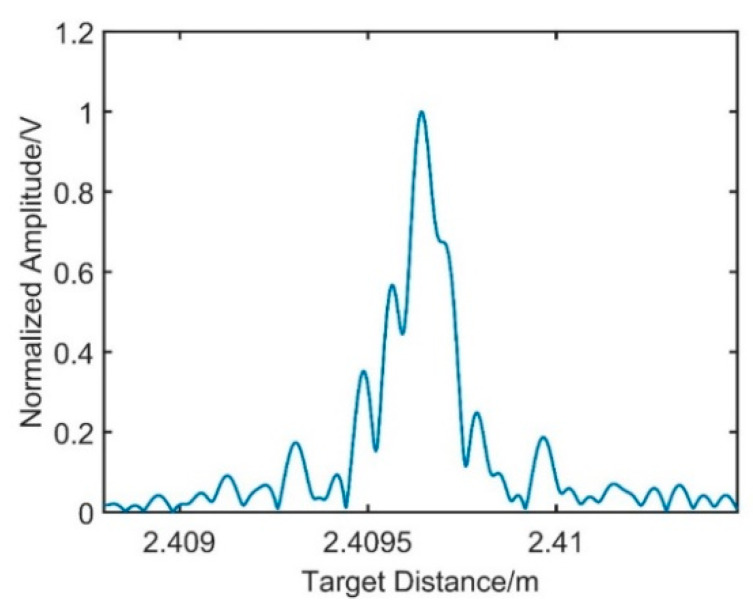
The distance spectrum of the resampling target spatial signal.

After the resampling signal passes through the linear phase band-pass filter, signal 3 and signal 4 are achieved, respectively. In Figure 7, the resampling target spatial spectrum can be obtained by phase operation with the Hilbert transform. The FWHM of the target spatial signal is 128 μm; compared with the FWHM of signal 3, it is broadened seriously, which will reduce the measurement resolution.

**Figure 8:**
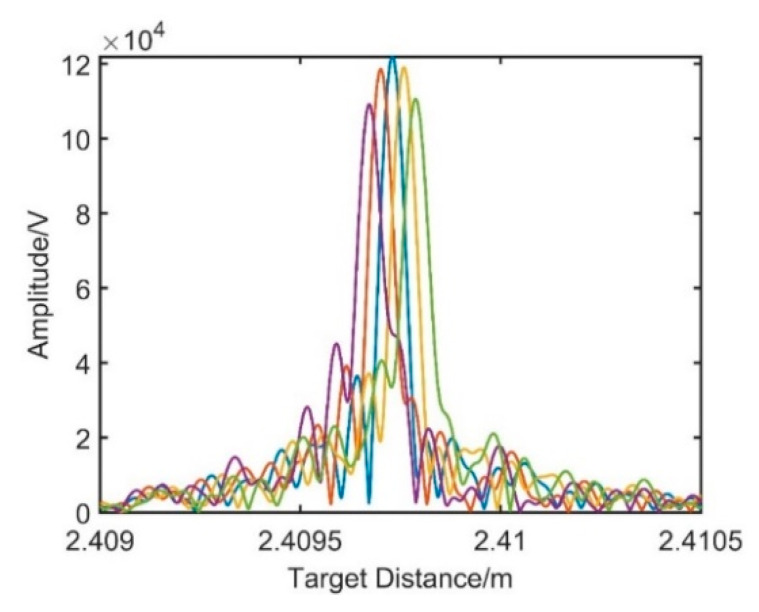
Dispersion compensation under different modulation coefficients.

In order to eliminate the dispersion mismatch between the two interferometers, a modulated signal whose phase has a proportional relationship with that of the target spatial signal should be constructed first. According to Equation (10), the reflected signal of the delay fiber 2 end face can be modulated and then sample the target spatial ranging signal as the reference clock. The modulation coefficient is determined by the evolution of peak variation, and the optimal modulation coefficient is determined based on the amplitude of the new resampling target spatial signal. Figure 8 shows the distance spectra obtained by resampling the target spatial signal under different modulation coefficients.

In Figure 8, from left to right, the modulation coefficients of the distance spectra are set to $-1.04\times 10^{-12}$, $-2.04\times 10^{-12}$, $-3.04\times 10^{-12}$, $-4.04\times 10^{-12}$, and $-5.04\times 10^{-12}$ in turn. As the modulation coefficients decrease, the phenomena of under compensation and overcompensation appear in the process. When the modulation coefficient of $-3.04\times 10^{-12}$ is taken, the amplitude reaches the maximum value. 

**Figure 9:**
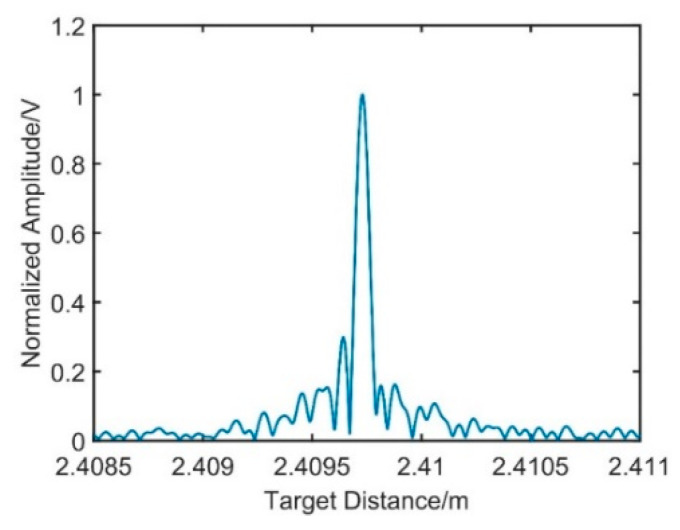
Dispersion compensation based on resampling of the modulated signal.

**Figure 10:**
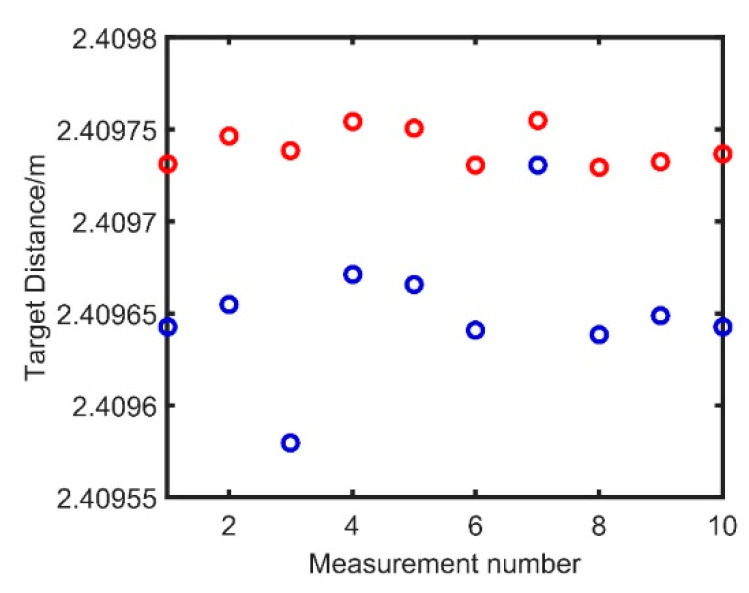
The results before and after the dispersion compensation.

In Figure 9, the FWHM of the target spatial distance spectrum reaches 73 μm, and it is the same with the FWHM of signal 3, which is not affected by the dispersion mismatch. In Figure 10, the target at the distance of 2.4 m is measured 10 times, where the blue points are the spatial distance before the compensation and the red points represent the distance obtained after the dispersion compensation. The standard deviation (STD) before compensation is 37.34 μm, and it is 10.12 μm after the compensation.

**Figure 11:**
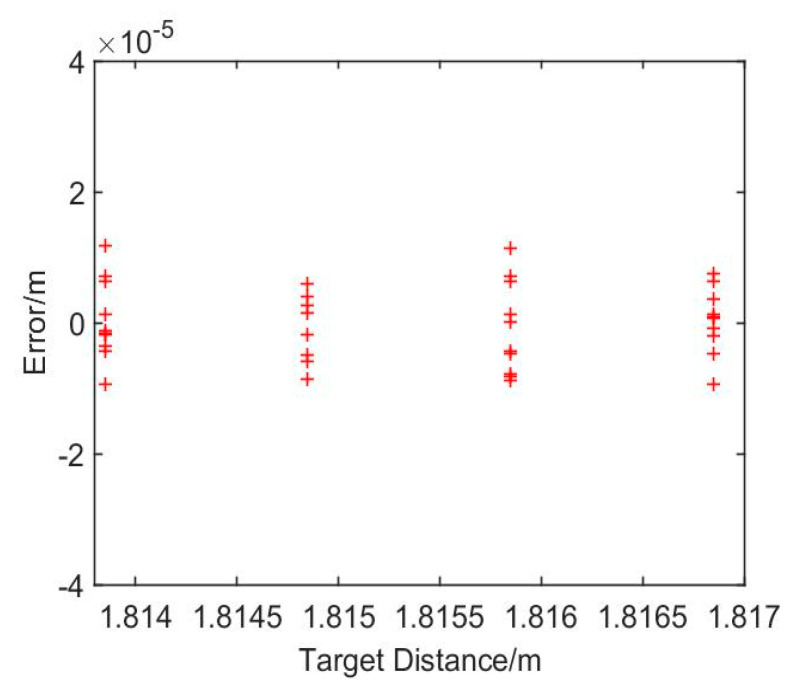
The results of different distances after dispersion compensation.

In addition, the targets at different distances are measured with this method. In order to make the displacement of the target coincide with the laser propagation path, a 1.8 m long guide rail is used to adjust the propagation direction of the laser. In the experiment, the collimator and the focusing lens are put on the guide rail, and the target is moved by a high precision Hexapod Motion with the step of 1 mm. In this experiment, five distances are measured, and the average of the results for the first distance is taken as the initial distance. The results of the rest four distances are shown in Figure 11.

In this picture, it can be seen that the distance errors after dispersion compensation are within 20 μm. As the ambient temperature changes, the OPD of the reference interferometer will no longer be a constant, which will affect the extraction of the equal phase intervals in the resampling process and then bring errors to the ranging results. In addition, the noise from the acquisition of the detector and the reflection process of the laser will affect the phase of the measurement signal, which will influence the phase extraction and phase calculation during the data processing. Moreover, the weak vibration of the optical platform in the external environment will also bring errors to the measurement of the target distance.

## 4. Discussion

The positive or negative of the modulation coefficient is related to the scanning direction of the modulated laser. When the laser frequency decreases, the modulation coefficient is negative, and the modulation coefficient is positive when the laser frequency increases gradually. In order to reduce the influence of noise on the reflected resampling signal of the delay fiber 2 end face (signal 3), a single frequency signal can be directly constructed according to the reference signal to replace it.

## 5. Conclusions

In a dual-interferometers system, the dispersion mismatch between the fiber reference interferometer and measurement interferometer will influence the system ranging. In this paper, a dispersion compensation method based on the resampling of the modulated signal is proposed. Since the reflected signal from the end of the delay fiber is not affected by dispersion mismatch, it can be modulated to generate a signal whose phase is proportional to the target spatial signal. Take the modulated signal as the new reference clock, and the influence of dispersion mismatch is eliminated. Experiments show that after modulated signal resampling, the FWHM of the new resampling target spatial signal is reduced from 128 μm to 73 μm. It is consistent with the FWHM of the reflected resampling signal from the end face of delayed fiber 2, which is not affected by the dispersion mismatch. A target at 2.4 m is measured, and the standard deviation of measurements is reduced from 37.34 to 10.12 μm. In this paper, the theoretical analysis and the experiment process are described, and the results show that the method proposed can eliminate the influence of the dispersion mismatch on the distance measurement, and it could provide a feasible reference in the high-precision target distance measurement.

## Figures and Tables

**Table 1:** Simulation parameters setting.

Initial Frequency of Laser (Hz)	The First Term Coefficient of μ(t)(Hz/s)	The Quadratic Coefficient of μ(t)(Hz/s)	Modulation Period (s)	The OPD of Delay Fiber 1 (m)
$193.54\times 10^{12}$	$6.3\times 10^{11}$	$4.35\times 10^{10}$	2	100
$v_{g}(m/s)$	$\beta_{2}(s^{2}/m)$	The OPD of Delay Fiber 2 (m)	Spatial Distance of the Target (m)	Sampling Frequency (MHz/s)
$2.0437\times 10^{8}$	$-23\times 10^{-27}$	10	6.5	8

## Data Availability

Not applicable.

## References

[1] Zhaoyu L., Chunfeng G., Zhaoyin W. (2019). Basics and developments of frequency modulation continuous wave LiDAR. Opto-Electron. Eng..

[2] Guang S., Fumin Z., Xinghua Q. (2014). Absolute distance measurement by high resolution frequency modulated continuous wave laser. Acta Phys. Sin..

[3] Swinkels B.L., Bhattacharya N., Wielders A. (2005). Absolute distance metrology for space interferometers. SPIE Opt. Metrol..

[4] Coe P.A., Howell D.F., Nickerson R.B. (2004). Frequency scanning interferometry in ATLAS: Remote, multiple, simultaneous and precise distance measurements in a hostile environment. Meas. ENCE Technol..

[5] Yang H.J., Nyberg S., Riles K. (2006). High-precision Absolute Distance Measurement using Dual-Laser Frequency Scanned Interferometry Under Realistic Conditions. Nucl. Inst. Methods Phys. Res. A.

[6] Cabral A., Rebordao J. (2005). Absolute distance metrology with frequency sweeping interferometry. Proc. SPIE.

[7] Minfu Z., Xinghua Q., Shenghua Y. (2008). Multiple sensor fusion in large scale measurement. Opt. Precis. Eng..

[8] Shi G., Zhang F., Qu X. (2014). High-resolution frequency-modulated continuous-wave laser ranging for precision distance metrology applications. Opt. Eng..

[9] Fouche D.G., Daniel G. (2003). Doppler imaging with dual-detection full-range frequency domain optical coherence tomography. Detection and false-alarm probabilities for laser radars that use Geiger-mode detectors. Appl. Opt..

[10] Asaka K., Ohbayashi K. (2007). Dispersion matching of sample and reference arms in optical frequency domain reflectometry-optical coherence tomography using a dispersion-shifted fiber. Opt. Express.

[11] Lippok N., Stephane C., Nielsen P. (2012). Dispersion compensation in Fourier domain optical coherence tomography using the fractional Fourier transform. Opt. Express.

[12] Lu C., Liu G., Liu B. (2015). Method based on chirp decomposition for dispersion mismatch compensation in precision absolute distance measurement using swept-wavelength interferometry. Opt. Express.

[13] Chundao S., Fumin S., Hao P. (2018). Dispersion correction in large-length range finding of frequency modulation continuous wave (FMCW). J. Infrared Millim. Waves.

[14] Xinke X., Guodong L., Bingguo L. (2015). High-resolution laser frequency scanning interferometer based on fiber dispersion phase compensation. Acta Phys. Sin..

